# Experiences of student nurses regarding the bursary system in KwaZulu-Natal province, South Africa

**DOI:** 10.4102/hsag.v24i0.1103

**Published:** 2019-02-28

**Authors:** Eve Jacobs, Belinda Scrooby, Antoinette du Preez

**Affiliations:** 1 School of Nursing Science, North-West University, South Africa

## Abstract

**Background:**

During 2010, the South African nursing education system was restructured, changing student nurses from having supernumerary status to being bursary holders. Changes with the introduction of this new bursary system included institutional factors and benefits that could be removed from the students, potentially hampering students’ sense of belonging.

**Aim:**

This study aimed to describe the experiences of students receiving bursaries in KwaZulu-Natal (KZN) province and to make recommendations for improving the system to bursary providers, educational institutions and practical settings based on these students’ experiences of the bursary system.

**Setting:**

The experiences of student nurses regarding the bursary system are described within a specified setting comprising two nursing campuses in KZN.

**Method:**

A qualitative study design was used and seven focus group interviews were conducted with purposively selected participants, representing the target population of first-, second- and third-year male and female nursing students registered for the Diploma in Nursing (General, Psychiatric, Community) and Midwifery.

**Results:**

Two main themes and eight subthemes were identified. The findings indicated that some of the bursary system’s experiences were negative as opposed to students having supernumerary status. These experiences had negative socio-economic, psychological, clinical, academic and family impacts. Many concerns related to staff members’ attitudes, shortages of nurses and service demands during students’ clinical practice assignments.

**Conclusions:**

The bursary system was not viewed as being beneficial to students as they did not receive all the benefits from being bursary holders. Support in clinical and academic areas was lacking as they were considered to be employees during their clinical assignments. There is an urgent need to review the bursary system.

## Introduction

In South Africa, nurse educators are challenged to prepare and equip student nurses for swift entry into the professional workforce to help address the dire shortage of nurses in the country (Wildschut & Mqolozana 2008:7). Developments in the Department of Health (DOH) of KwaZulu-Natal (KZN) province of South Africa necessitated changes in the funding of the education and training of student nurses. Since 2010 the student nurses in KZN province rely on bursary payments for financial support and, as a result, might have to take on extra jobs to support themselves (Duffin & Waters 2005:15). McCarey, Barr and Rattray (2007:358) stated that students might have to pay back debts if they are unsuccessful in completing their training. Rochford, Connolly and Drennan (2009:601) indicated that in the traditional model of nursing education, student nurses were paid employees of the relevant training hospital. The DOH in KZN province used to treat nursing students as employees (with a personnel number), paying them a monthly stipend with all-inclusive packages as part of their bursaries (Torerai 2012:23). The total bursary per year of study is R30 000 for the enrolled programme and R36 000 for the 4-year diploma course. This indicates that the student nurses in the enrolled programme receive a monthly stipend of R2500 and the 4-year diploma student nurses an amount of R3000 without additional benefits. The students need to purchase textbooks, uniforms, meals and pay for their accommodation from this amount. An increase in bursary funding according to Montgomery, Tansey and Roe (2009:35) could alleviate financial problems and decrease the financial pressure on students.

Student nurse numbers appear to be declining, which might be attributed to the financial crises related to student nurses’ bursaries (Palese et al. 2012:e60).

Cuthbertson, Lauder, Steele, Cleary and Bradshaw (2004:380) questioned the role that the bursary system plays in recruiting student nurses. Their research indicated that the students experienced increased insecurity, weakening the nursing education system. Some student nurses commence their training in their late twenties, when they have families with demands on finances and time. If these students do not have sufficient money to pay their bills, they will work additional hours to support their families (Duffin & Waters 2005:15).

Steele et al. (2005:576) agreed that student nurses encounter challenges concerning financial difficulties, household and childcare responsibilities and, being mature students, problems of balancing work, family and academic life. Steele et al. (2005:576) considered student nurses’ financial difficulties to be a prominent concern.

Malone and Robertson (2006:27) suggested that student nurses with bursaries should be trained in a well-supported and respected framework. Student bursaries should be sufficient to enable the students to support themselves to pay for childcare facilities. Wright and Maree (2007:607) stated that some hospitals rely on students as a workforce. According to Bowden (2008:45), student nurse attrition rates increase, and thus the effects of the bursary system on students’ experiences should be investigated.

### Problem statement

Having students on employee status and remunerated accordingly in 2007 proved too expensive for the DOH (Breier, Wildschut & Mgqolozana 2009:85) of KZN province. Thus the bursary system was implemented. During 2010 nursing education was restructured in KZN province, with the KZN College of Nursing (KZNCN) changing students from having supernumerary status to being bursary holders. The Democratic Nurses’ Organisation of South Africa (DENOSA) protested against the bursary system for student nurses as the benefits of the bursary system were less than those of the previous salary-based system (DENOSA 2012:1). Students would find it difficult to buy food, uniforms and textbooks and demanded that the previous salary-based system of R162 000.00 per year for education and salary costs should be reinstated (Kimberley 2012:2). Democratic Nurses’ Organisation of South Africa stated that by implementing the bursary system, the DOH was now ignoring the poorest of the poor and aggravating student attrition rates (Kimberley 2012:2). The current study’s findings could help to clarify the bursary students’ experiences and to address identified challenges, possibly also reducing the student nurse attrition rate.

Hamshire, Willgoss and Wibberley (2012a:2) explored factors influencing student nurses’ attrition rates. Student nurses most frequently mentioned financial concerns (33%), compared to academic dissatisfaction (26%) and clinical placement problems (14%). According to Hamshire et al. (2012a:3), finances impacted on student nurses’ academic and clinical achievements. Some students worked at shops over weekends to earn more money and thus had limited family time. Such difficulties made students question whether it was worthwhile continuing with their nursing studies (Hamshire et al. 2012a:6).

In order to address this problem, the research question was as follows:

What are the experiences of student nurses who receive bursaries in KwaZulu-Natal province?

### Aim and objectives

Based on the above research question, this study aimed to:

- describe the experiences of students receiving bursaries in KZN,
- make recommendations for improving the system to bursary providers, educational institutions and practical settings based on these students’ experiences of the bursary system.

### Conceptual definitions

The following definitions represent the core concepts applicable to this article:

*Bursary* - The bursary scheme was implemented to reduce the cost for the DOH for student nurses’ training in KZN. This includes the total amount awarded to a student nurse on commencement of training. According to the DOH (2010:68) it is divided into 12 monthly payments and paid directly into each student’s bank account.

*Experiences* - These are the conscious events that make up an individual’s life (Merriam-Webster Dictionary 2018). In this article, it is how the student nurses experienced the bursary system in KZN.

*Student nurse* - This refers to an individual undergoing training in basic nursing (DOH 2008:5), while Breier et al. (2009:13) define student nurses as those following the 4-year nursing programmes either at universities or colleges. In this article student nurses were those who were registered for the 4-year Diploma in Nursing (General, Community, Psychiatric) and Midwifery at two nursing colleges in KZN.

## Research design

The study used a descriptive qualitative (eclectic) and contextual research design.

## Research method

### Population

The target population comprised first-, second- and third-year student nurses on the bursary system at two KZN Nursing College campuses.

### Sampling

Non-probability, purposive sampling was used. Male and female student nurses could participate in the study provided that they received bursaries and were in the first, second or third year of the 4-year nursing programme. The fourth-year student nurses were not on campus (as they were in their psychiatric hospital rotation during the data collection period); moreover, fourth-year students had supernumerary status (and did not receive bursaries) and were thus excluded from participating in this study.

### Data collection

Seven focus group interviews were used to gain a detailed picture of the student nurses’ experiences regarding the bursary system in KZN and also to follow up on interesting avenues emerging during the interviews (Botma, Greeff, Mulaudzi & Wright 2010:208).

Interviews were conducted between February and March of 2014 at the two KZN nursing campuses, at settings where the participants (P) were comfortable, and their voices were audio-taped. Face-to-face data collection took place. The participants were asked to provide chronological verbal descriptions of their experiences as bursary holders. The seven focus group interviews lasted 25–30 min. A single broad question was used: ‘Tell me about your experiences of being a bursary student?’

Recordings were transcribed verbatim and text results were analysed (Botma et al. 2010:214). Comprehensive field notes assisted with data analysis. The typed field notes were attached to each transcription.

### Data analysis

The verbatim transcriptions of the interviews were analysed and encoded using a content analysis technique. A consensus discussion was held by the researcher and an independent co-coder and a decision was reached about the main themes and the subthemes that emerged from the written text, in order to ensure trustworthy coding (Brink et al. 2012:194).

### Ethical considerations

Permission to conduct the study was granted by the ethics committee of North-West University, Potchefstroom Campus (Reference: NWU-00144-13-A1), and the KwaZulu-Natal Department of Health (KZNCN). Classrooms were appropriated for interviews as they were comfortable and private. The first author explained the purpose of the study and obtained informed consent from each participant to audio record the interviews prior to commencement of each interview.

## Rigour

The researchers used the framework of Lincoln and Guba (1985:290–311), which is supported by Field and Morse (2002:1–19) and Botes (1995:143–147), namely truth value (credibility), transferability (applicability), consistency (dependability) and neutrality, also known as conformability.

Credibility was achieved through prolonged engagement (as clinical accompanist) with the participants to establish trust. Transferability was achieved through the purposeful selection of the participants (1–3-year bursary students), as well as through a dense description of the research methodology and results of the study. Dependability was achieved through a detailed description of the research design, method and context and the inclusion criteria for purposive, voluntary sampling. Confirmability was achieved through seven focus group interviews, field notes, data analysis and the interpretation of themes and subthemes. Similar findings were obtained by all listed research methods.

## Findings and discussion

Seven focus group interviews were conducted until data saturation had been achieved. Two main themes and eight subthemes were identified.

## Theme 1: Experiences

### Subtheme 1.1 - Socio-economic aspects

Many students described how living costs, childcare and medical expenses caused financial strain on a daily basis.

‘It is not sufficient enough to cater for all the needs, especially for a student.’ (Focus group 7, Participant 11, Campus B)

Hanson (2014:1) explained that in order to facilitate students’ progress their physiological needs, including food, shelter, clothing and sleep, should be met. Freitas and Leonard (2011:12) agreed that the faculties have a responsibility to assist students to meet their needs. Resources should be adequate and available in order to encourage improving the status of nursing education and training (DOH 2008:17).

Students indicated that they had families and children to support on their stipends. Cuthbertson et al. (2004:374) also found that students’ financial and childcare problems could lead to attrition. Andrew et al. (2008:869) claimed that students experienced difficulties managing family and health finances.

‘When you have a small child, there are more expenses towards that. So if you don’t get paid for six months, how are you going to survive? It’s disposable nappies, they need milk, they need medical attention.’ (Focus group 7, Participant 12, Campus B)

Missing 6 months’ training because of pregnancy left a student without money; she had to raise a newborn child without money and possibly without proper food. Students could not understand why the previous system included maternity benefits while the bursary system did not do so. Maternity leave, as stated in the KZNCN rule book (2005:10), is considered to be an interruption of training and is thus exclusive of all benefits.

Bursary status students were not compensated for any overtime during their clinical assignments, yet were expected to work night duty, weekends and public holidays.

**TABLE 1:** Themes and the subthemes as identified.

Themes	Subthemes
Theme 1: Experiences	Subtheme 1.1: Socio-economic aspects Living costs and expenses – food, transport, rent, books, clothes, uniforms Childcare – school fees, clothes, transport, living expenses, single parents, nanny, day care Medical – maternity leave, injuries, communicable diseases Overtime not paid Register for SARS Subtheme 1.2: Psychological Sad Frustrated Angry Psychologically draining Tired and exhausted Feel inferior Punishment Overworked Intimidated Embarrassed Discouraged Feel exploited, being used Stressed Have no voice Scared to be expelled Guilt when falling pregnant Feel threatened Subtheme 1.3: Expectations From educational institution – academic achievements, working hours during weekends, night duty and holidays From family – support financially, childcare, single parents, relationships with spouse From practical settings – to help with workload, part of workforce, work like permanent staff, clinical demands, work out of scope of practice, shortage of staff, patients’ needs Subtheme 1.4: Effects Dropouts Abortions Stealing and theft Injuries, risks and illnesses Dating sugar daddies Leave other jobs to come and study nursing because of passion and love for nursing Not enough time for academic work and studying Not best choice of academic institution, because receive bursary not at all institutions; therefore not always best place to study Relationships with family – spouse’s expectations as well as have to get money from family for financial support Have debt Have to change lifestyle Extended studies, for example, when falling pregnant Risk of being sued, no legal covering Subtheme 1.5: Positive Have a job when finished Sometimes rent lower because receive bursary, for example, pay only half of rent Educational expenses not so expensive compared to other study fields and institutions Grateful to become a nurse Motivation to work hard so as not to lose bursary
Theme 2: Recommendations	Subtheme 2.1: Bursary provider Increase incentives, money for books, uniforms, transport, stipend for living expenses Give benefits, for example, maternity leave, medical aid, pension, transport, accommodation, meals, books, uniforms, stationery Openness and transparency about what bursary includes or does not include Return to supernumerary status Subtheme 2.2: Educational institution Transport to and from work Limit costs of prescribed books Meals and accommodation – nursing home dirty Enough equipment for teaching and learning Recreational area Support – emotional and physical Extend library hours so it can be used after work Subtheme 2.3: Practical setting Give medical treatment where working Treat as students, not as permanent staff – workload

SARS, South African Revenue Service.

Students faced hardships to cope without maternity leave benefits, medical aid or compensation for injuries.

### Subtheme 1.2 - Psychological

Hamshire, Wilgoss and Wibberley (2012b:185) agreed that negative experiences during clinical placements could influence students’ decisions to discontinue their training. Students raised concerns that they sometimes felt humiliated during their allocated times in the wards, causing them to consider abandoning their training.

‘I mean you get this hatefulness from the trained sisters or the other staff nurses, which I do not know where it comes from and then … from my personal point, I’ve come to a point where I said, I want to resign.’ (Focus group 1, Participant 1, Campus A)

Students raised concerns about permanent staff members’ attitudes, which impacted on students’ experiences during clinical placements. Levett-Jones, Lathlean, Higgins and McMillan (2009:319) also reported that students said that unfriendliness, resentment and hostility of nursing staff members made them feel uneasy.

‘Some of the trained staff actually makes us look like we are dumb.’ (Focus group 1, Participant 8, Campus A)

Student nurses often experienced high levels of stress during training that might result in psychological or emotional impairment during their professional lives, ultimately affecting the quality of patient care provided (Shaban, Khater & Akhu-Zaheya 2012:204).

Students experienced emotional and social difficulties that impacted on their participation during training (Steele et al. 2005:576). Job dissatisfaction levels among nurses contributed in two distinct ways to reducing the quality of patient care. Dissatisfied nurses with lower morale were more likely to leave, aggravating nursing shortages and increasing nurse turnover rates, while the performance of nurses might be adversely affected by low motivation levels (Horwitz & Pundit 2008:24).

‘It’s really, uh, depressing I’d say, and it’s, it makes you lose focus because now you end up focusing on money and how much you’re getting [*rather*] than really focusing on what you came here to do.’ (Focus group 1, Participant 3, Campus A)

‘You find that there are so many patients that are complaining about the nurses’ behaviour. They say that the nurses, they treat them badly. How can you go to the ward without satisfaction. … You take your anger and the only person that you see is the patient. It puts the patients’ lives at risk.’ (Focus group 4, Participant 16, Campus A)

Feelings of frustration, disappointment and of being taken for granted were common, as many participants explained that the stakeholders failed to provide the required support to the students. The academic requirements and the clinical workloads caused anxiety.

Factors such as anxiety, health status, psychological stress and economic stability according to Freitas and Leonard (2011:9) impacted on clinical and classroom performance. Warbah et al. (2007:600) stated that psychological distress, poor adjustment and coping skills could result in poor academic performance among students, which could contribute to attrition. The students verbalised the following:

‘… Very, very disappointed and frustrated when it comes to the bursary system. It makes us feel as if we are being taken for granted.’ (Focus group 2, Participant 10, Campus A)

‘I feel like I’m being exploited. Especially since what other people were getting before. Why can’t I get [*that*], I am doing exactly the same as everyone but I’m not getting anything at all.’ (Focus group 6, Participant 4, Campus A)

It is evident that the demands on student nurses were high and they did not feel part of the nursing team, which could produce psychological effects.

### Subtheme 1.3 - Expectations

Student nurses might have had misconceptions regarding nursing as a profession and about the required academic knowledge (Wright & Maree 2007:597). Student nurses’ academic expectations, according to Hamshire, Wilgoss and Wibberley (2013:173), might be based on irrelevant information. The expectations of students in the current study will be discussed from three different points of shared experiences.

### Expectations from the educational institution

Students felt overloaded and clinical placements affected their ability to complete assignments timeously. Hamshire et al. (2012a:2) agreed that students might enter the profession to find that there are very high academic course expectations.

Several students shared their reasons for failing to meet educational expectations:

‘… our campus where we study, the problem is that, we are also called bursaries, but we go to the wards from 7 to 7 some of the days, and then we are also supposed to study for college works, which makes me feel we are overloaded because we sometimes, not even sometimes, most of the times you cannot study if you go to the ward, you work hard, you stand from the morning until noon and then you are supposed to go back and study and that is the cause of failure to the bursary students.’ (Focus group 4, Participant 17, Campus A)

Another student nurse referred to the demands from the lecturers and the workload that they were expected to meet during clinical placements:

‘Tutors in the college, they also want their assignments, they want us to pass the test, which [*means*] they also give us loads of work. We study from the morning until four o’clock and then also it is also overloaded the students.’ (Focus group 3, Participant 8, Campus A)

### Expectations from family

Brodie et al. (2004:728) mentioned that students needed to balance work demands and family life, especially as student nurses are often older than other students. Duffin and Waters (2005:15) added that students’ family needs must be met in order to succeed in their training and to be effective at work.

‘In my family it is just me and my mom and we have to take care of about seven people. So with the little that have, with the little that I get from this bursary system, I can’t afford to do that.’ (P12)

### Expectations from practical settings

Brodie et al. (2004:729) raised concerns about students who were not coping with their courses because of excessive work demands. Duffin and Waters (2005:14) stated that studying nursing might not warrant any favours that are not extended to other students. However, the nature of nurses’ training is different as they have to work extended hours, weekends and during holidays. The bursary students regarded themselves primarily as students requiring clinical learning experience. However, they perceived themselves to be treated as full-time employees, required to meet work demands.

‘We are forced to work, we are working full shifts, seven to seven like other workers.’ (Focus group 2, Participant 13, Campus A)

‘But … it makes me very angry at the same time because they are actually using us to basically do what they are employed to do, whereas we are still students but we are not treated as students; they treat us as employees but they don’t give us the same money that the employees actually get.’ (Focus group 6, Participant 6, Campus A)

### Subtheme 1.4 - Effects

Student nurses desired to live more manageable lives like other staff members without the need to take on inappropriate tasks to earn extra money.

The effects of the bursary system could have negative impacts, as mentioned by students:

‘… a sugar daddy comes up, you will jump for it and some of us have actually jumped for sugar daddies.’ (Focus group 6, Participant 2, Campus A)

‘I have also been tempted to get myself a sugar daddy because you’ve got someone who is willing to provide. You are so broke, you will actually settle for anything.’ (Focus group 6, Participant 4, Campus A)

The reality is that some student nurses resorted to ‘sugar daddies’ to meet their academic, personal and clinical needs.

Student nurses who had left other jobs to pursue nursing careers regretted these decisions, which could influence the attrition rates because of financial hardships (Wright & Maree 2007:597).

‘… we are regretting, we [*are*] even wishing to quit because we are not satisfied with the bursary system..’ (Focus group 4, Participant 14, Campus A)

‘Before I came here I was an employee. When I came here I thought I was going to get something better, but at the same time I regret [my decision]. I even think to, to [*submit a*] resignation letter because I’ve got things to do, I’m a breadwinner at home and my daughter is in DUT.’ (Focus group 1, Participant 6, Campus A)

Students claimed that advertisements were misleading because they started the course without proper directives and information. Students suffered as a result of leaving their other jobs and then made the shocking discovery of the bursary amount.

### Subtheme 1.5 - Positive attributes

Some students expressed positive attributes about their clinical learning experiences. Students shared that some of their best and most rewarding moments in the clinical area were associated with patients’ appreciation for students’ work. Feelings of joy and of being appreciated, according to Crombie, Brindley, Harris, Marks-Maran and Thompson (2013:1286), could actually make students feel better about themselves. The manner in which the students were treated and received in the clinical areas had major impacts on why they actually remained in the profession (Crombie et al. 2013:1286). Being bursary students motivated them to be conscientious during practical placements in order to retain their bursaries. The students shared the following about their rewarding and motivating experiences:

‘[*The*] best situations are in the wards with the patients, where you will get someone who is grateful that you have done something good to them.’ (Focus group 1, Participant 1, Campus A)

‘Being a student in the bursary system motivates me to work even harder because you can lose your place. As soon as you [*come*] in, you can lose it. So uh, it motivates me to be more careful and study harder.’ (Focus group 7, Participant 16, Campus B)

## Theme 2: Recommendations

Theme 2 identified students’ recommendations for better options with regard to the bursary system.

### Subtheme 2.1 - Bursary provider

The bursary provider should improve the bursary system and funding. Supernumerary status, with salaries and benefits, would enable students to be self-supporting, especially as they needed to juggle family life, financial issues and their studies. One student stated:

‘It would have been better, if maybe they [*increased*] the pay, maybe we would have been able to meet the ever-increasing demands.’ (Focus group 7, Participant 12, Campus B)

### Subtheme 2.2 - Educational institution

The students said that shortage of equipment, materials and staff impacted negatively on their clinical learning experiences.

‘The place where we practise our practicals, there [*is*] less equipment so we end up doing things that we are not supposed to be doing due to shortage of material. So it would be good if they can plan us [*an*] adequate place where you could get everything because we end learning things that are wrong.’ (Focus group 5, Participant 19, Campus B)

‘… we don’t have the backup of the college, because you find that in the wards they are short of staff and they would take students from whichever group to cover that shortage of staff.’ (Focus group 4, Participant 18, Campus A)

As nursing colleges are usually close to teaching hospitals, student nurses were often required to provide services (Ali & Naylor 2010:160), to address staff shortages, while attending college. This reduced students’ time to study.

### Subtheme 2.3 - Practical setting

Each institution is accredited for specific clinical facilities for student nurses to obtain their 4000 required clinical hours in specified clinical areas during their 4-year programme, as stipulated by the South African Nursing Council (SANC) (KZNCN 2004:6). Students felt that the practical environment was threatening and detracted from their acknowledgement as students, causing despondency.

‘… even if you are 5 minutes late, we are actually treated very badly and they tell us “remember you are bursary students, if you are not back we will start an abscondment”.’ (Focus group 6, Participant 4, Campus A)

‘Some sisters say, I told you not to leave and then you [*left*] the ward so I’m going to put this day as absent, and then you will owe the hours and pay them back.’ (Focus group 4, Participant 9, Campus A)

## Limitations

The study was conducted at two nursing college campuses in KZN that are affiliated to public hospitals for clinical practice. Hence the study’s findings are limited to the public environment in KZN province only.

## Recommendations

Sufficient numbers of permanent trained staff should be employed to prevent students from being used to provide service, thus failing to meet students’ expectations in the clinical environment. Lecturers must strengthen clinical teaching to promote and enhance more competent trained nurses. Bursary holders experienced clinical and theoretical learning experiences to be compromised as clinical service needs superseded student learning needs. Further research should be done to investigate whether the bursary system contributes to problems such as the declining status of nursing, attrition of student nurses and the rate of student nurses’ absenteeism.

## Conclusions

Factors pertaining to the bursary system could have negative impacts on students, affecting their abilities to meet their basic needs, family demands, academic requirements, clinical work assignments and workload. Being treated as full-time employees, rather than full-time students, impacted negatively on the bursary holders’ clinical and theoretical learning experiences, attributable to the requirements of working overtime without remuneration.
